# Giant recurrent left inguinal hernia with femoral nerve injury: a report of a rare case

**DOI:** 10.1186/s12893-020-00786-9

**Published:** 2020-06-09

**Authors:** Manzhou Lin, Guojie Long, Ming Chen, Weice Chen, Jian Mo, Nianping Chen

**Affiliations:** Department of Hernia and Abdominal Wall Surgery, Affiliated Hospital of Guangdong Medical University, Guangdong Medical University, Zhanjiang, China

**Keywords:** Giant inguinal hernia, Recurrent hernia, Nerve injury, Inguinal hernia repair

## Abstract

**Background:**

Giant inguinal hernia(GIH), a rare disease, has brought great challenges to surgeons. GIH is defined as an inguinal hernia that extends below the midpoint of the inner thigh in standing position. However, a giant recurrent inguinal hernia resulting from previous operations that destroy the anatomical structure of the inguinal region is extremely rare. Nerve injury, a complication following inguinal hernia repair, is mostly found in ilioinguinal nerve and iliohypogastric nerve, which often presents as numbness and acute or chronic pain, while postoperative muscular dysfunction results from femoral nerve injury is rare.

**Case presentation:**

A 77-years-old woman presented with a complaint of a reducible mass in the left inguinal of duration 1 year. The patient had three previous inguinal hernia repairs. Physical examination and auxiliary examination indicated a giant inguinal hernia with femoral nerve injury. After preoperative evaluation and preparation, a transabdominal partial extraperitoneal(TAPE) repair have performed. Finally, the patient recovered and was discharged.

**Conclusions:**

In conclusion, we reported a rare case of a giant recurrent inguinal hernia with femoral nerve injury and made a successful treatment for the patient via transabdominal partial extraperitoneal(TAPE) repair.

## Background

**Fig. 1:**
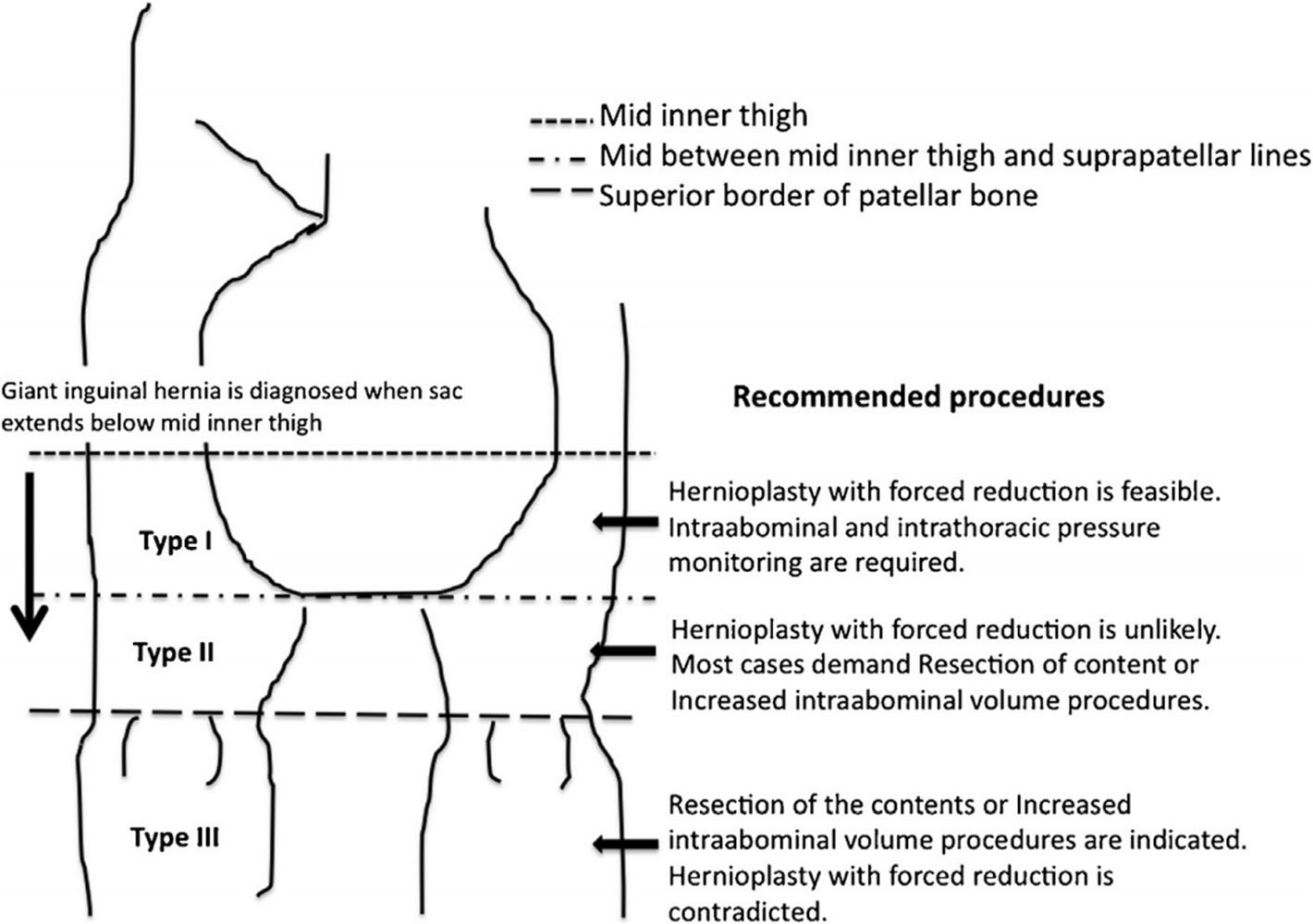
New classification of giant inguinal hernia and recommended procedure (by Trakarnsagna et al., 2014, modified)

Inguinal hernia is a very common disease. However, giant inguinal hernia(GIH), a rare disease, has brought a great challenge to surgeons. GIH appears when patients neglect the treatment for many years and it is defined as an inguinal hernia that extends below the midpoint of the inner thigh in standing position [1]. However, a giant recurrent inguinal hernia resulting from previous operations that destroy the anatomical structure of the inguinal region is extremely rare. New classification of GIHs and recommended procedures was suggested by Trakarnsagna et al. in 2014. They categorize giant inguinal hernia into three types, depending on the location and options for surgical operations [2] (Fig. 1). Nerve injury, a complication following inguinal hernia repair, is mostly found in ilioinguinal, iliohypogastric, and the genital branch of the genitofemoral nerve, which often presents as numbness and acute or chronic pain [3], while postoperative muscular dysfunction results from femoral nerve injury is rare.

In this article, we reported a rare case of a giant recurrent inguinal hernia with declined muscle function caused by femoral nerve injury.

## Case presentation

A 77-years-old woman presented with a complaint of a reducible mass in the left inguinal since 1 year. The patient was diagnosed as an indirect left inguinal hernia by Abdominal CT at the local hospital. The patient had a history of 3 inguinal hernia repairs. All three repairs were open herniorrhaphy and a mesh was placed in the previous third operation, unfortunately, the remaining details were not well clear.

**Fig. 2:**
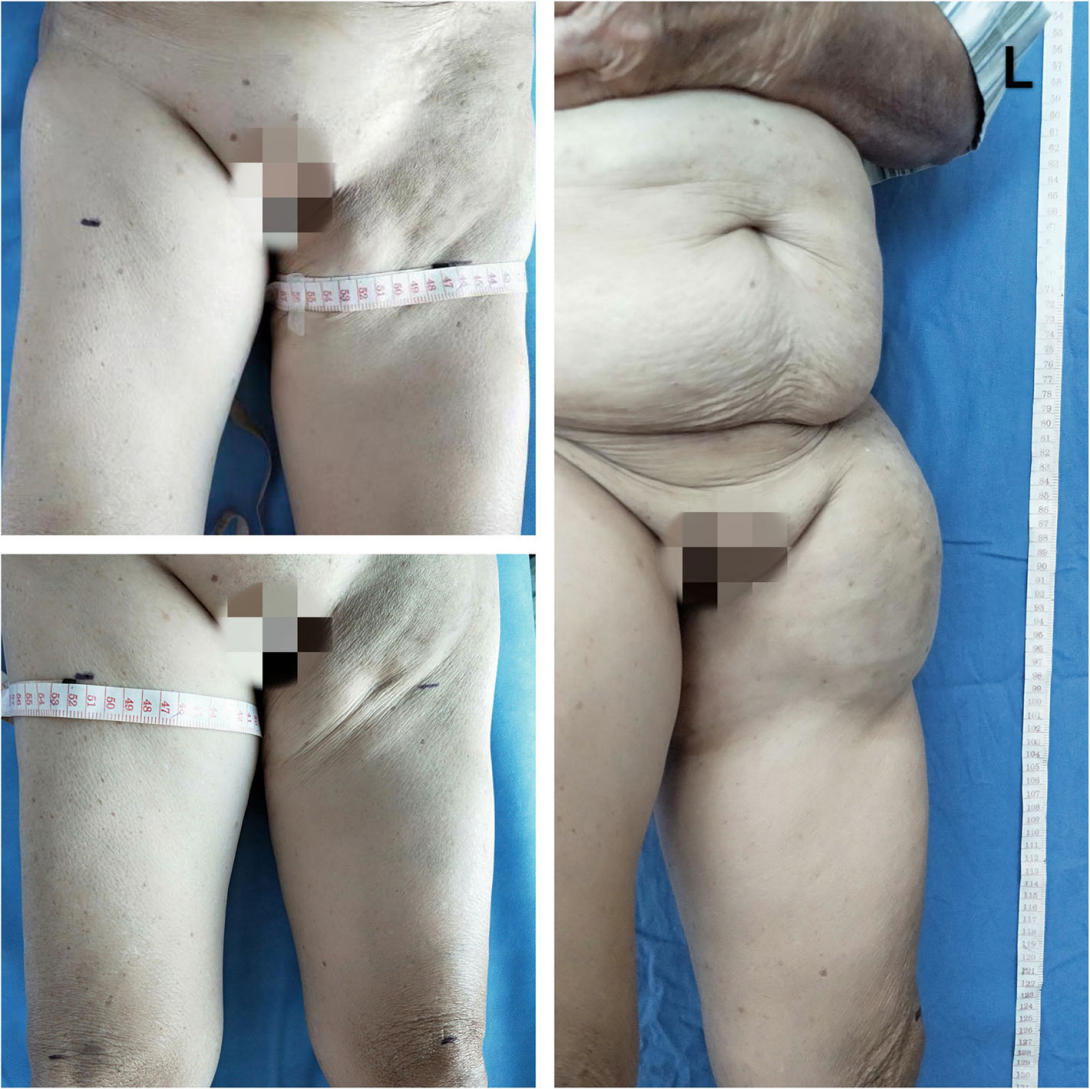
Contrast of both thighs while standing

Physical examination after admission: The blood pressure was 220/110 mmHg, the remaining vital signs were normal. An about 10 cm surgical scar was seen in the left inguinal area. A reducible mass in the left inguinal area was detected, which could drop in standing and coughing. The mass can reach the midpoint of the thigh when it drops to its maximum size(18 cm × 14 cm). The circumference of the left and right thighs is unequal(Left:47 cm vs Right:52 cm, Fig. 2), The anteromedial sensation of the left thigh was lost, the sensation of the right side was normal. The bilateral knee-extensor motor was normal. The contraction test of sartorius muscle was positive on the left side and was negative on the right side, and the patellar reflexes, achilles tendon reflexes, and plantar responses of both lower limbs were normal.

**Fig. 3:**
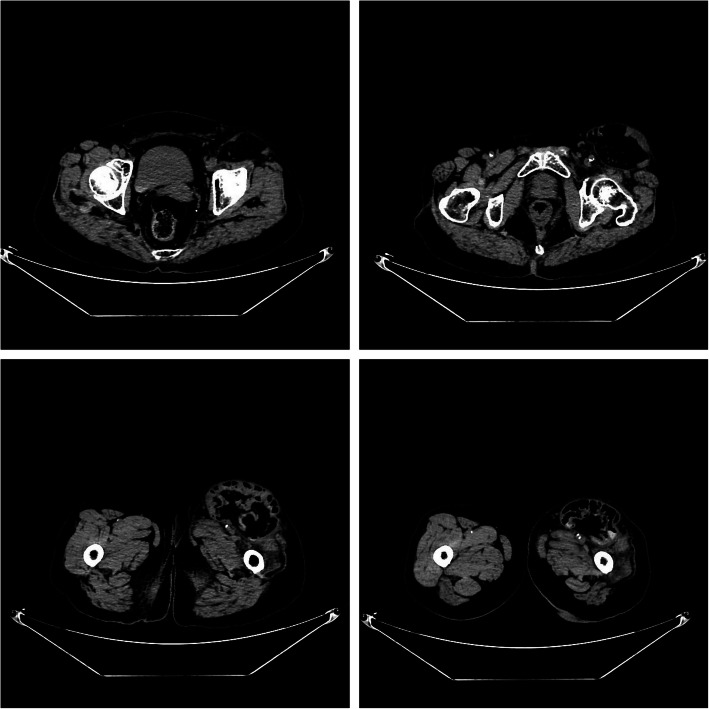
CT images of the lower abdomen and Thighs

**Fig. 4:**
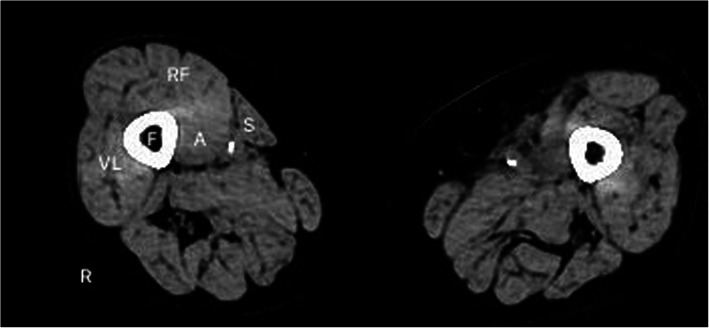
CT cross-section image at the level of the upper thigh reveals atrophic changes of the rectus femoris, adductor muscle, and sartorius muscle on the left. Normal side is labeled for identification of structures:S = sartorius muscle, A = adductor muscle, RF = rectus femoris, VL = vastus lateralis, F = femur

**Fig. 5:**
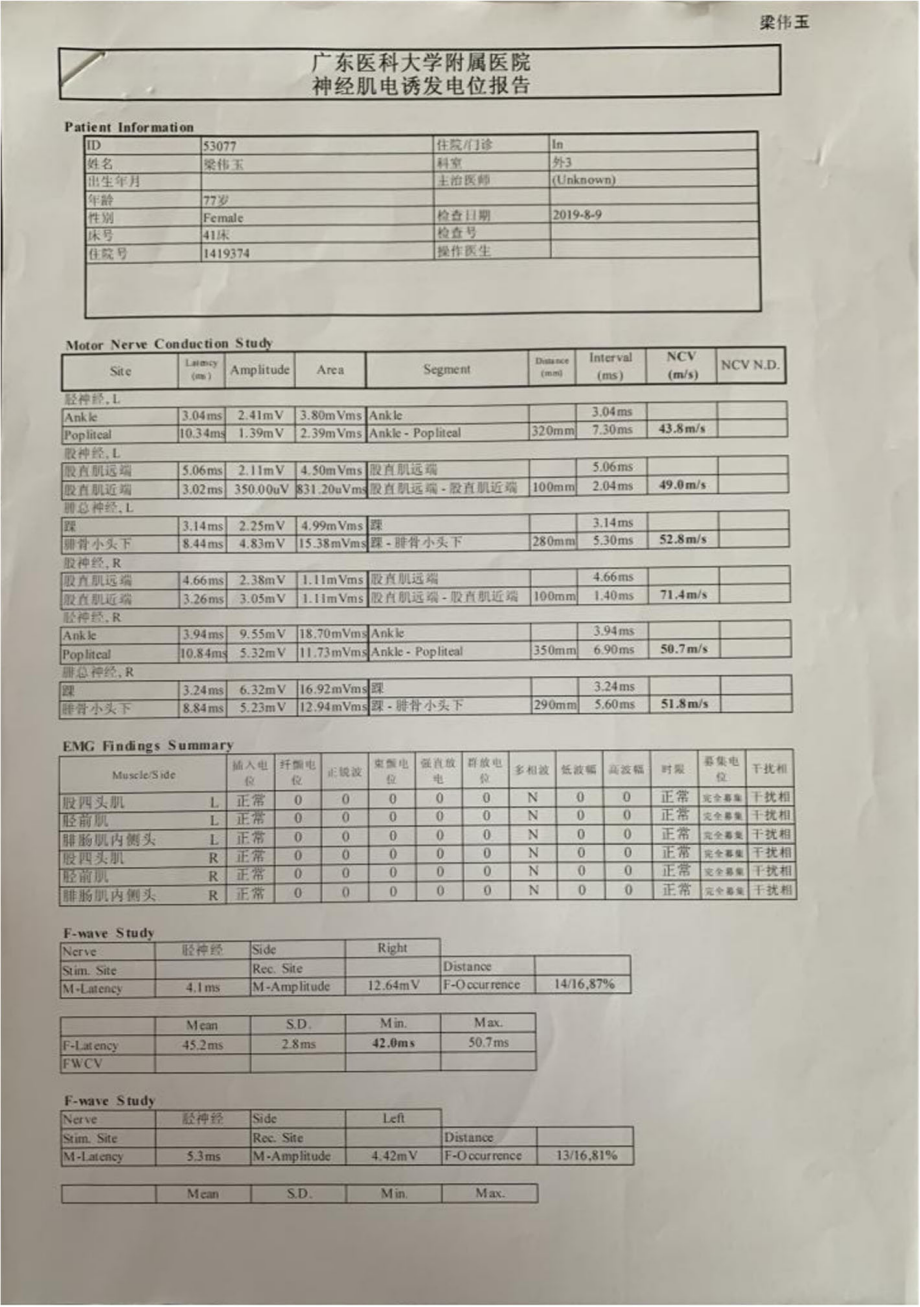
The No.1 picture of the EMG and NCV

**Fig. 6:**
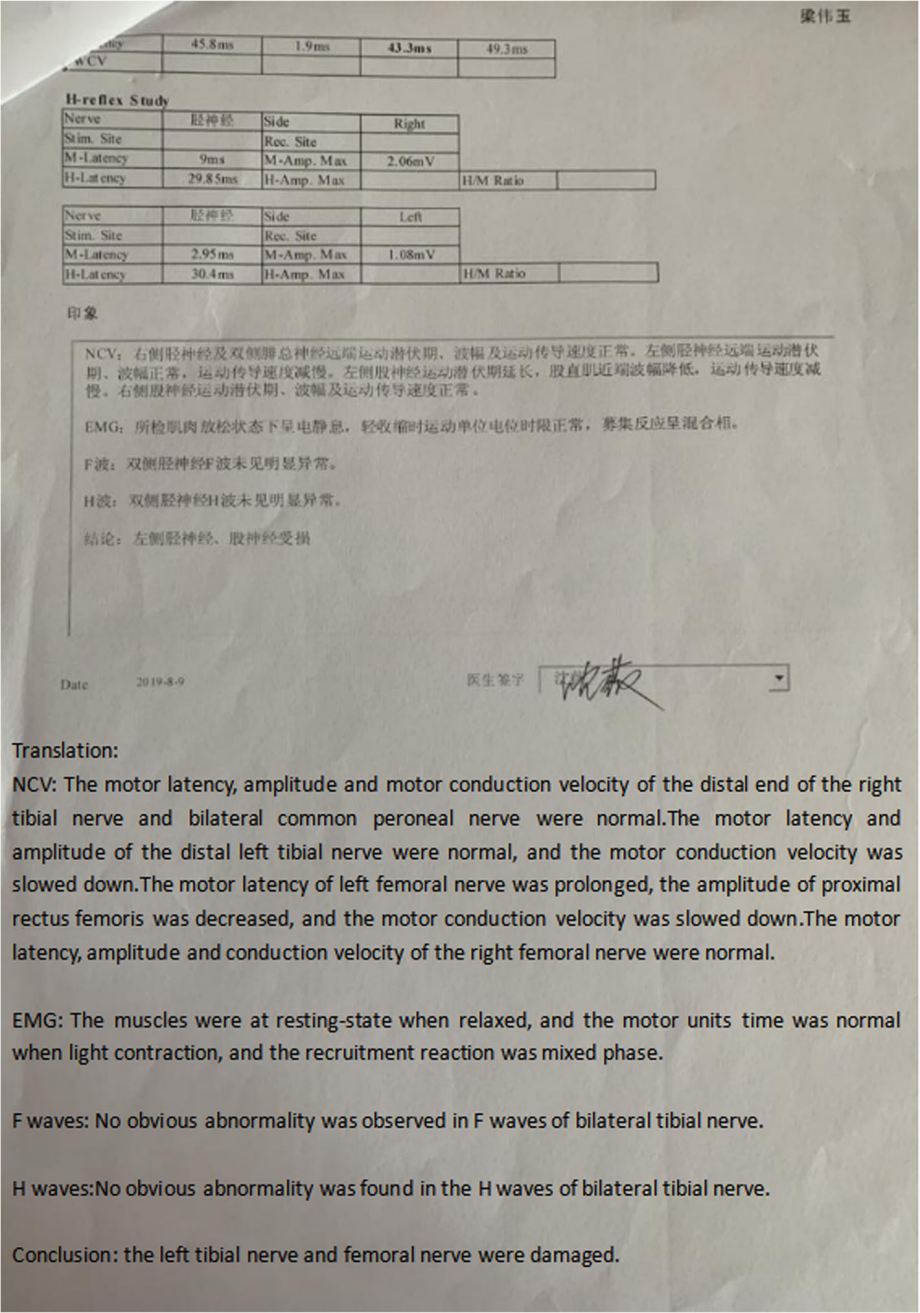
The No.2 picture of the EMG and NCV including translation of the conclusion

After admission, relevant laboratory and imaging examinations were completed: blood routine examination and coagulation function examination were normal. Abdominal ultrasound finding suggested that the diagnosis was left inguinal hernia. The results of the computed tomography (CT) conformed to an inguinal hernia (Fig. 3), the atrophy of rectus femoris, adductor muscle, and sartorius muscle were visible in the CT scan (Fig. 4). Also, electromyography(EMG) and nerve conduction velocity(NCV) of the lower limbs showed the reduction of conduction velocities with prolongation of motor latency of left femoral nerve and reduced proximal rectus femoris amplitudes, indicating that the left tibial nerve and femoral nerve were damaged (Figs. 5 and 6).

**Fig. 7:**
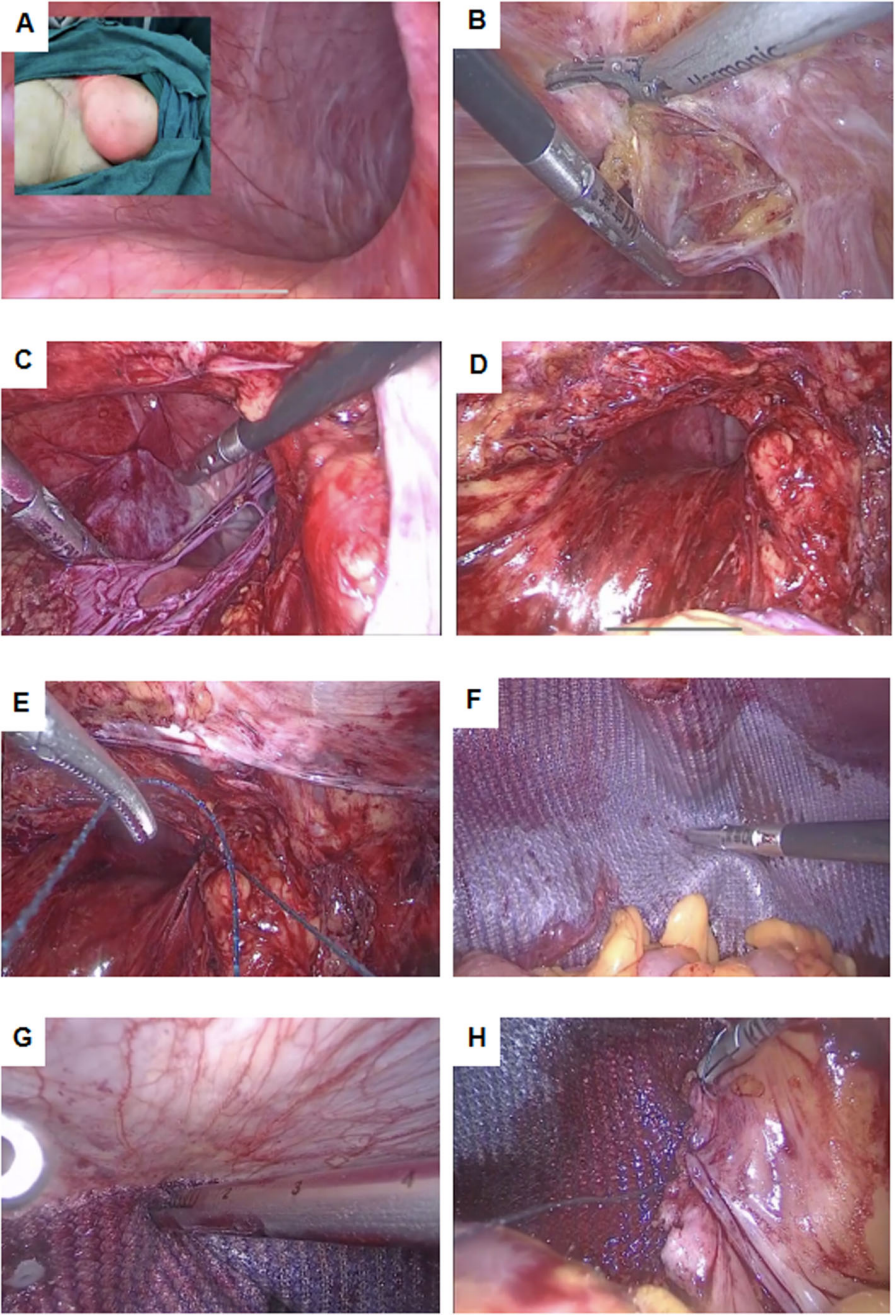
Intraoperative findings of left-sided giant inguinal hernia. **a** Abdominal exploration and giant hernia orifice. **b** Dissection of the peritoneum. **c** Dissection of the hernia sac. **d** After dissection of the preperitoneal space. **e** The distal hernia sac opening was sutured. **f** The prosthetic mesh placed on the hernia orifice. **g** The mesh was fixed by absorbable tacks on its upper edge. **h** The mesh was fixed by suture on its lower edge

After the preoperative preparation, we selected a laparoscopic pre-peritoneal technique because of three open surgery histories. The patient was positioned supine, underwent surgery under general anesthesia and indwelling catheterization. The laparoscope was inserted through a 10-mm umbilical port. Surgical exploration showed a few focal adhesions, omentum which is entrapped inside a hernia sac and a hernia orifice whose size was 6 cm × 5 cm. Another 10-mm port was placed at the lateral edge of the right abdominal rectus muscle and a 5-mm port was placed at the lateral edge of the left abdominal rectus muscle. Following lysis of the adhesions, the content in the hernia sac was reduced by pulling the contents carefully using forceps. And then an about 8 cm peritoneal incision was made superiorly concerning hernia orifice and the boundaries of which was 2 cm beyond the medial and lateral sides of the hernia orifice. The hernia sac was dissected, a negative pressure drainage tube was placed in distal hernia sac(between the mesh and the transversalis fascia), and the opening of hernia sac was closed by continuous self-locking suture. At that time, we wanted to complete the extraperitoneal repair, but the peritoneum could not be closed due to the high tension. Finally, a 20 × 15 cm bioresorbable coating mesh that comes from BARD Corporation was placed to cover hernia defect and fixed by BARD absorbable tacks on its upper edge and by suture on its lower edge. The operation time is 110 min, the Intraoperative bleeding volume is 10 ml and none of the complications occurred during the operation (Fig. 7).

**Fig. 8:**
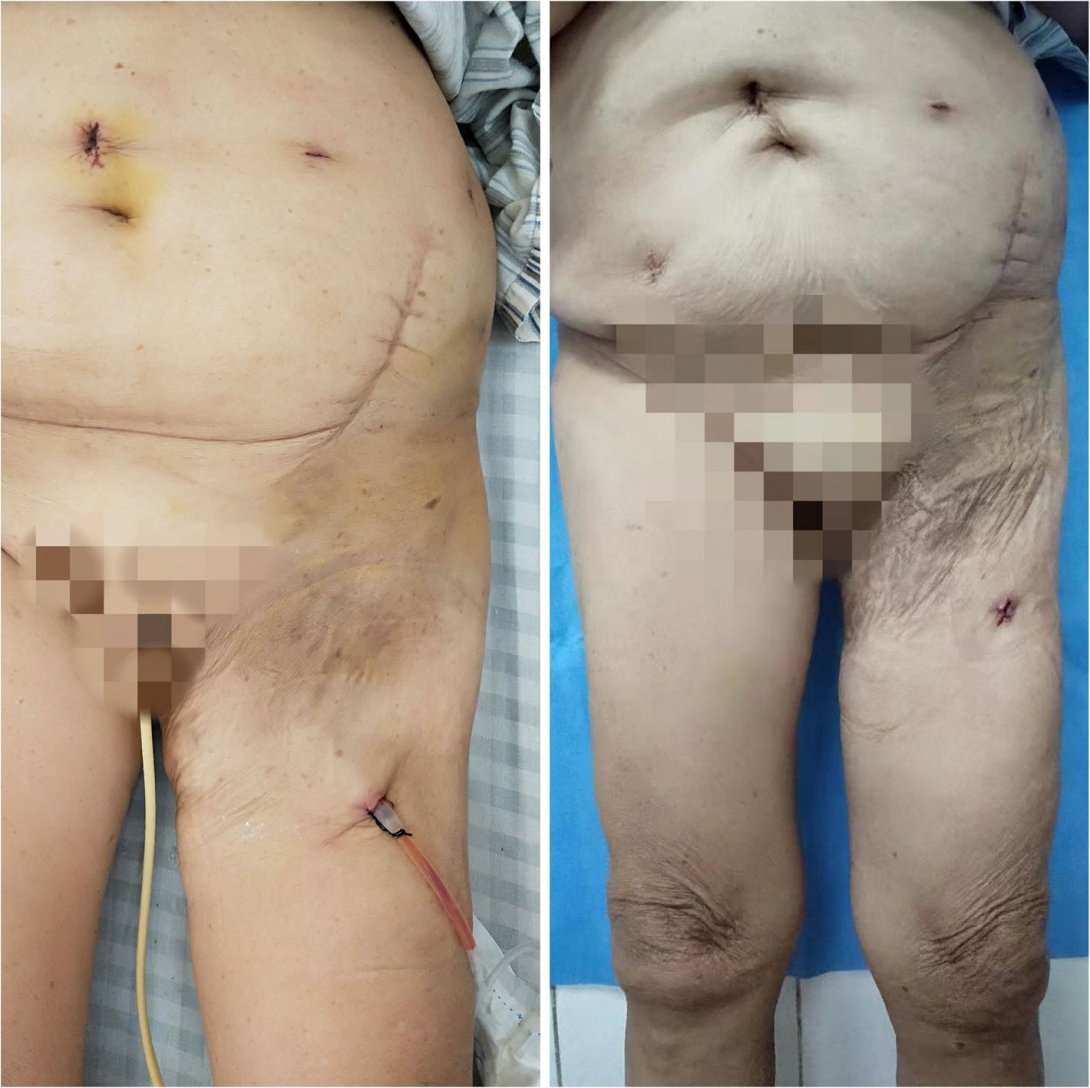
Postoperative photos. One negative pressure drainage tube and one urine tube were indwelled postoperatively. The negative drainage tube was subsequently removed 72 h after the surgery

In the final, the drainage tube was subsequently removed 72 h after the surgery and the patient was discharged from the hospital on the tenth postoperative day. At the time of patient discharge, the anterior medial skin sensation of the patient’s left thigh recovered a little and the sartorius contraction test was still positive (Fig. 8). As a result of the COVID-19 pandemic, the recovery of the femoral nerve is not known without physical examination and auxiliary examination, but the patient did not experience recurrence over the six-months telephone follow-up period.

Although the results of EMG and NCV suggested that patients still had left tibial nerve injury, the patient did not have the relevant clinical manifestations of tibial nerve injury. So the authors thought that the evidence of tibial nerve injury was insufficient, so we did not discuss the tibial nerve injury in this paper.

## Discussion and conclusion

The femoral nerve comes from the lumbar plexus from l2 to l4 and passes down through the muscular space in the deep surface of the iliac fascia slightly lateral to the middle point of the inguinal ligament into the femoral triangle and is scattered into several branches. These branches supply the extensor muscles and the skin of the anterior and medial aspects of the thigh and leg. The 2–3 branches innervate the sartorius muscle. A complete femoral nerve injury would present as paralysis of the quadriceps, hypoethesia of the anterior and medial thigh and leg, and loss of the patellar reflex [4].

At present, Cases of femoral nerve injury following inguinal hernia repair are rarely reported in the literature. Several cases of femoral nerve injury following inguinal hernia repair have been described [5–7]. Burning or stabbing pain in the inguinal area and anterior thigh was the predominant initial symptom in those cases. Other signs and symptoms include quadriceps weakness presenting as difficulty with extension movement of the knee, altered sensation in the sensory distribution of the nerve, and atrophy of the quadriceps muscle. In this case, the clinical presentation of femoral nerve injury was atypical, included anterior compartment group muscle atrophy, loss of sensation of the anterior and medial thigh, and dysfunction of the sartorius muscle, but was not pain. The unequal circumferences of both thighs, CT images, EMG, and NCV can all support the diagnosis of femoral nerve injury. The clinical manifestations of femoral nerve injury differ due to variations in the location of damage, and The branches innervating the sartorius muscle were probably damaged in this case.

Although no cases of nerve injury due to compression of giant inguinal hernia have been reported in the literature, this cause cannot be excluded. From the CT image, we can see that it is the location of the hernia that shows muscular atrophy. Unfortunately, we cannot identify the cause of femoral nerve injury in this case, because the details of the previous three repairs are unclear and time of injury is unknown due to the patient’s unawareness of symptoms. However, we think the compression of hernia is more likely because the clinical manifestations are more like chronic injury caused by nerve entrapment. Under those circumstances, the damage to the nerve is considered to be irreversible.

There is some statement regarding how to deal with this complication. In cases where pain is the only symptom, conservative treatment is the treatment of choice—for example, painkillers, physiotherapy, or selective nerve blockade [3]. If pain is the only symptom, a surgical revision should be reserved for those patients who do not respond to non-surgical pain management treatment for 3 months [8]. And neurectomy of the damaged nerve has been described in the literature with favorable outcomes [9]. Haninec et al. think that an immediate surgical revision should be performed in patients with concomitant motor dysfunction [10]. For patients with severe symptoms that affect daily living and ineffective conservative treatment, timely surgical treatment should be chosen.

In three previously reported cases, all patients received surgical treatment. In one of three previously reported cases were the nerve directly injured by a suture piercing it [6]. In the other two cases there was stretching and compression of the nerve by scar tissue. In each case freeing of the scar tissue by careful neurolysis at exploration led to immediate relief of pain and a more gradual improvement in function. The persistent finding was atrophy in one case, which was unimproved after 4 months [5]. In this case, the patient suffered from motor dysfunction, which was an indication for the surgery but didn’t affect her daily living. And the patient’s intention is primarily to deal with the giant hernia. In consequence we didn’t perform any further surgical exploration of the damaged femoral nerve.

According to our experience, we provide recommendations to avoid nerve injury in inguinal hernia repair: The layers of anatomy in an operation should be clear. Blind dissection, non-dissection, and minimal dissection are not recommended. Don’t try to dissect the nerves, but protect the nerves you can see in the surgical field. In cases of nerve transection during surgery, immediate reconstruction is the only possible treatment. Traction injuries caused by excessive retractor tension or compression are usually detected after surgery. These cases usually respond well to conservative treatment.

The repair of GIHs has been discussed in the literature previously. To avoid the development of abdominal compartment syndrome, resulting from a sudden elevation of the intraabdominal pressure following organ reposition, the preoperative administration of progressive pneumoperitoneum therapy or components separation of the abdominal wall to enlarge the abdominal space was suggested [11]. What about open or laparoscopic? Open abdominal and inguinal approaches are commonly used. Several authors reported that the transabdominal preperitoneal prosthetic(TAPP) approach for GIHs resulted in recurrences and emphasized its limitations. Since one case in their study was a giant bilateral scrotal hernia, it might have been difficult to reduce, and an open approach might have been needed for the resection of the abdominal contents [12]. Fortunately, the hernia in this patient can be reduced by manipulation, so we are not concerned about abdominal compartment syndrome caused by organ reposition. The case is also a recurrent inguinal hernia. The European Hernia Society guidelines described that laparoscopic recurrent inguinal hernia repair is recommended after failed anterior tissue or Lichtenstein repair [13]. According to the classification of recurrent inguinal hernia, this case is classified as R3 and laparoscopic pre-peritoneal technique is recommended [14]. Based on the considerations above, we opted for a laparoscopic pre-peritoneal procedure. Because the peritoneum was very thin and the tension was very high, we could only put part of the mesh outside the peritoneum and part inside the abdominal cavity(transabdominal partial extraperitoneal, TAPE).

Seroma is a common complication after GIHs repair, especially in cases where the hernia sac has not been completely dissected. So a negative pressure drainage tube was placed in the remaining hernia sac in this case.

In conclusion, we reported a rare case of a giant recurrent inguinal hernia with femoral nerve injury and made a successful treatment for the patient via transabdominal partial extraperitoneal(TAPE) repair.

## Data Availability

The datasets used and/or analysed during the current study are available from the corresponding author on reasonable request.

## Abbreviations

GIH: Giant inguinal hernia
TAPE: Transabdominal partial extraperitoneal
CT: Computed tomography
EMG: Electromyography;
NCV: Nerve conduction velocity
TAPP: Transabdominal preperitoneal prosthetic
